# Generalized arterial calcification of infancy with a novel *ENPP1* mutation: a case report

**DOI:** 10.1186/s12887-018-1198-4

**Published:** 2018-07-05

**Authors:** Iole Brunod, Barthélémy Tosello, Sophie Hassid, Catherine Gire, Laurent Thomachot, Michel Panuel

**Affiliations:** 1 Pediatric and Neonatal Intensive Care Unit, Hôpital Nord, Assistance Publique-Hôpitaux de Marseille, Chemin des Bourrely, 13015 Marseille, France; 2 Department of Neonatology, Hôpital Nord, Assistance Publique des Hôpitaux de Marseille (APHM), Chemin des Bourrely, 13015 Marseille, France; 3 Aix Marseille Univ, CNRS, EFS, ADES, Marseille, France; 4 Department of Medical Imaging, Assistance Publique-Hôpitaux de Marseille, Hôpital Nord, Chemin des Bourrely, 13015 Marseille, France

**Keywords:** *ENPP1*, Generalized arterial calcification of infancy, Arterial calcification, Neonatal hypertension

## Abstract

**Background:**

Generalized Arterial Calcification of Infancy (GACI) is a heritable ectopic mineralization disorder resulting in diffuse arterial calcifications and/or stenosis, mostly caused by mutations in the *ENPP1* gene. Here we present a case report of GACI in a male infant with a new familial mutation of the *ENPP1* gene and the clinical outcome after biphosphonates therapy.

**Case presentation:**

The clinical presentation was characterized by a severe early-onset of hypertension refractory to multiple therapy. To investigate this atypical hypertension, a renal Doppler ultra-sonography was performed and diffuse echo-bright arteries were detected; then a low-dose whole-body computed tomography demonstrated extensive arterial calcifications, suggesting GACI. A novel homozygous mutation c.784A > G (p.Ser262Gly) was detected in the *ENPP1* gene. The infant was administered four courses of bisphosphonates: arterial calcifications were found to decrease but severe refractory hypertension was persistent*.* Although GACI can be a rapidly fatal illness and frequently results in death in infancy, the patient was 24 months of age at the time of writing this report.

**Conclusions:**

Three points of interest: the first one is to remind clinicians of this rare and atypical etiology in neonates with severe hypertension and in fetuses with cardiomyopathy and non-immune hydrops fetalis. The second point is the identification of a novel mutation in the *ENPP1* gene associated with a clinical presentation of GACI. The third point is the fairly favourable outcome of our patient after bisphosphonates therapy, with calcifications regression but not hypertension.

## Background

Generalized Arterial Calcification of Infancy (GACI, OMIM 208000), also known as idiopathic infantile arterial calcification, is a heritable ectopic mineralization disorder resulting in diffuse arterial calcifications [1] mostly caused by mutations in the *ENPP1* gene [2]. GACI is characterized by prenatal or infantile onset of widespread arterial calcifications and/or narrowing of large- and medium-sized arteries [3], without neither gender nor ethnic predilection [4]. Commonly, patients present with variable cardiovascular phenotypes and symptoms with onset in-utero or in the first months of life [4]. GACI is inherited in an autosomal recessive manner. The classic form of GACI is caused by mutations in the *ENPP1* gene (GACI1, OMIM 208000), localized to chromosome 6q22-q23 [5]. To date, therapeutic guidelines are lacking. Being synthetic analogues of inorganic pyrophosphate, the most reported GACI therapy are bisphosphonates: their efficacy is not completely proven as some case reports show inconsistent results and clinical trials are not feasible [3, 6]. Prognosis is poor; most affected individuals die within the first year of life from cardiovascular complications [4].

GACI is a rare disease; data about this condition, especially regarding clinical course and treatment efficacy, is mainly based on clinical series and case reports [4]. This case report of neonatal GACI contributes to enrich the literature with data on a new familial mutation of the *ENPP1* gene and provides details of the specific clinical course after biphosphonates therapy.

## Case presentation

The male patient was the naturally conceived son of a 34-year-old gravida 2, para 0, miscarriage 1 mother. His healthy parents were consanguineous (first-degree cousins, of Algerian origin), without history of hypertension, calcification or cardiomyopathy. Prenatal concerns included a fetal hypertrophic cardiomyopathy (HCM) associated with a hydrops fetalis and a polyhydramnios, treated by one amnioreduction due to poor maternal tolerance. No other abnormality was noted on the prenatal ultrasound. Furthermore, antenatal investigations of fetal cardiomyopathy were negative or normal (enzyme disorder, maternal viral infection, normal fetal karyotype). Preterm birth was medically indicated because of an abnormal fetal heart rate: the newborn was delivered by urgent caesarean section at 29 weeks’ gestation. The newborn was eutrophic (birth weight 1330 g), umbilical cord pH was 7.21; his Apgar scores were 4 at 1 min, 8 at 5 min, 10 at 10 min. Resuscitative and neonatal appropriate care were provided.

**Fig. 1:**
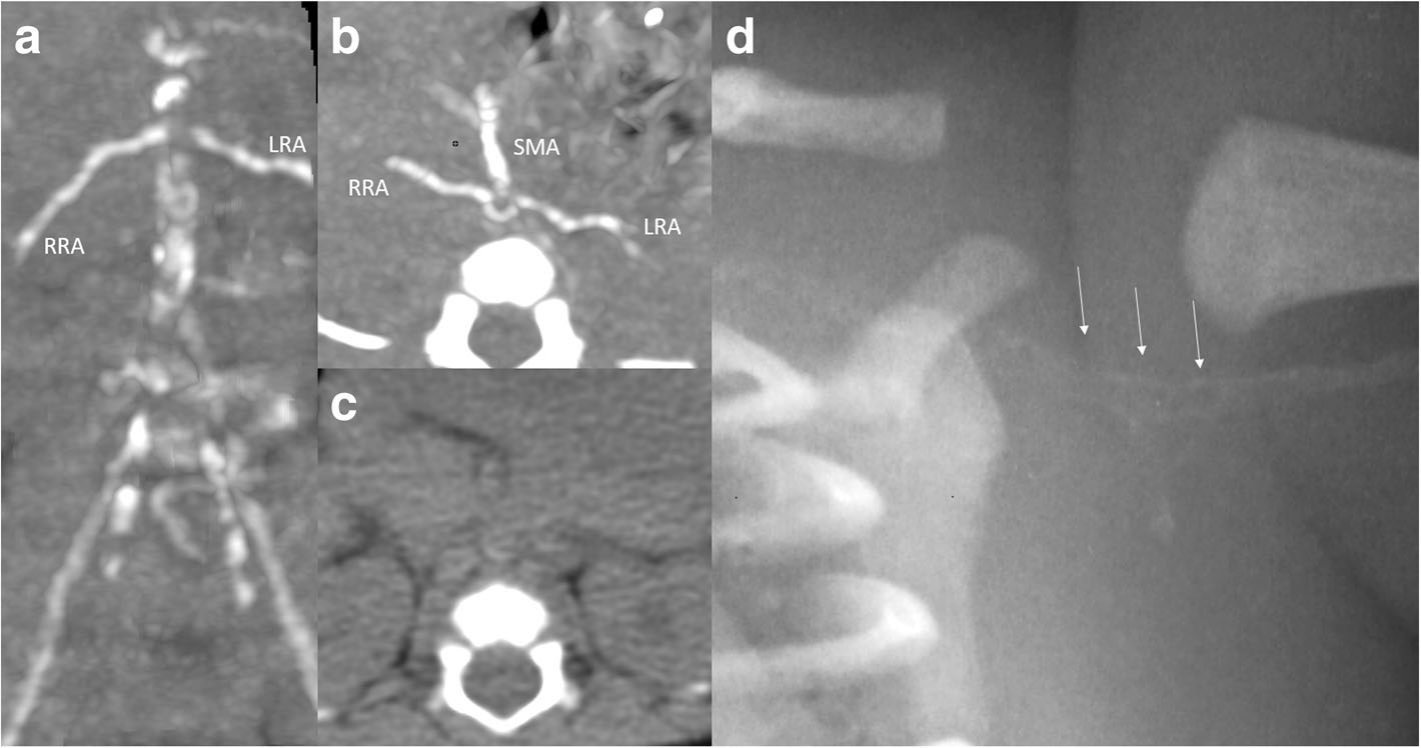
**a** Abdominal CT-scan frontal view at 1 month of age: huge vascular calcifications of the aorta, the iliac arteries, the renal arteries (RRA: right renal artery, LRA: left renal artery). **b** Abdominal CT-scan axial view at 1 month of age: calcifications of the superior mesenteric artery (SMA), renal arteries (RRA, LRA) and the aorta. **c** Abdominal CT-scan axial view at 5 months of age: the arterial calcifications are no longer visible. **d** Close-up view on the left scapular area of chest X-ray at day 1: subtle calcifications of the left subclavian and left axillary artery (white arrows)

At birth, he presented with hydrops fetalis (oedema and mild pericardial effusion); he was not dysmorphic; postnatal echocardiography confirmed the diagnosis of HCM without other heart anomalies and routine x-ray didn’t show bone abnormalities. The patient developed severe hypertension a few hours after birth (mean arterial pressure > 70 mmHg, normal range: 35–40 mmHg). This hypertension was refractory to a triple therapy (propranolol, nicardipine, clonidine). To assess this atypical hypertension, extensive blood and urinary tests (for endocrine disease and inborn error of metabolism) were performed, but yielded negative results. A renal artery Doppler ultrasound was performed and detected extensive calcifications of the renal arteries, the abdominal aorta and its major branches. To further elucidate the extent of the calcifications, a low-dose whole-body computed tomography was performed and detected diffuse calcifications of large- and medium-sized arteries (Fig. 1a, b). The brain Magnetic Resonance Imaging was normal at term equivalent age. In view of these findings we retrospectively reviewed the chest x-ray performed at day 1 of life: it is noteworthy that calcifications were already apparent (Fig. 1d). Prenatal ultrasound images were also retrospectively reviewed by several radiologist experts but no arterial calcifications were detected. These singularities were consistent with GACI: the patient’s genotype was analysed, and a mutation was found in the *ENPP1* gene*.*

The treatment consisted of four courses of bisphosphonates administered by intravenous route on day 40 of life, on day 70 of life, on month 21 of life and at 2 years old: each course consisted of 2 or 3 disodium pamidronate infusions (0.50 mg/kg/infusion) delivered over 1 week. At 20 week-follow-up visit, 2 months after the two initial courses of bisphosphonates, the extent of the arterial calcifications was assessed by a whole body computed tomography and an almost complete resolution of the lesions was demonstrated (Fig. 1c). Concurrently, from 2 months-old to one-year-old, the baby developed transiently hypophosphatemia (serum phosphate level of 3.1 mg/dL (1.0 mmol/L); normal infant range = 4.6–7.1 mg/dL and 1.5–2.3 mmol/L) with inappropriate urinary phosphate excretion (fractional excretion of phosphate > 5%). All other relevant markers of phosphate homeostasis were within normal range (serum 1,25-(OH)_2_-vitamin D level, parathyroid hormone level, serum calcium level, serum alkaline phosphatase level). The infant had no bone deformities. The transient hypophosphatemia was corrected with oral phosphate supplementation and no vitamin D supplementation. Concerning cardiovascular outcome, at 2-year-old follow-up, hypertension was still treated with a triple combination of drugs: blood pressure levels were constantly above 140/80 mmHg. However, hypertrophic cardiomyopathy resolved with this treatment. The patient had normal growth and development. He had neither ocular nor cutaneous manifestations of Pseudoxanthoma Elasticum (PXE).

The investigation of a multi-gene panel was performed by high-throughput next-generation sequencing. Genomic DNA was processed with the TruSight One/Nextera hybrid capture method and coding regions with adjacent intronic regions of the two GACI-associated genes *ENPP1* and *ABCC6* were enriched by PCR (Illumina^®^). Subsequent analysis was conducted by massive parallel sequencing using the MiSeq Benchtop System (Illumina^®^). Quality criteria require a minimal coverage of 20× for at least 97% of target regions of the investigated genes: the minimal coverage of 20× achieved for all targeted regions was 98% with an average coverage of 172 sequences per base. For assessment of the variants, the dbSNP, ExAc, HGMD, LOVD and UniProt databases, as well as the prediction programs PredictSNP, PhDSNP, SNAP, SIFT, PolyPhen-1, PolyPhen-2, MutationTaster, SNAP, MAPP and Panther were used. Next-generation sequencing analysis of the patient’s *ENPP1* gene revealed a homozygous mutation c.784A > G (p.Ser262Gly). There was no mutation in the *ABCC6* gene. The parents consented to undergo genetic testing. The c.784A > G (p.Ser262Gly) mutation was also present, in the heterozygous state, in the consanguineous parents. The mutation c.784A > G (p.Ser262Gly) has so far not been described in the literature and mutation databases: there are no entries for the variation in the 1000 Genomes project and the Exome Aggregation Consortium, which indicates a very low global minor allele frequency (MAF). The prediction programs PredictSNP, PhDSNP, SNAP, SIFT, PolyPhen-1, PolyPhen-2, MutationTaster and SNAP all classify the variation as pathogenic whereas the prediction programs MAPP and Panther classify the variation as benign.

The combination of cardiovascular findings and extensive calcifications on imaging, together with the *ENPP1* mutation, confirmed the diagnosis of GACI1 in the proband.

## Discussion

We report a case of neonatal GACI: clinical presentation was a severe early-onset hypertension, investigations found diffuse calcifications on imaging and a mutation in *ENPP1* gene. The *ENPP1* mutation found in the proband is not yet described in the literature. Even though the clinical presentation, results of investigation and management reported in this publication have been already described, outcome and clinical course after bisphosphonates are of interest. Indeed, biphosphonate is the most reported therapy but guidelines are lacking and results are not consistent, emphasizing the importance of collecting data: here, arterial calcifications were found to decrease (Fig. 1c) but severe refractory hypertension was persistent. Although GACI can be a rapidly fatal illness and frequently results in death in infancy, the patient was 24 months of age at the time of writing this report.

Diagnosis of GACI is made by the combination of clinical, imaging or histopathological findings, together with genetic results. The clinical presentation described in this case report was previously reported in the literature: hypertension was reported in 22% of patients and cardiomegaly in 10% of patients [4]. Cardiovascular phenotype is quite variable in GACI disease, including fetal distress, polyhydramnios, hydrops fetalis, heart failure, hypertension, cardiomegaly, visceral effusion, respiratory distress, cyanosis or reduced peripheral pulse [4]. Some case reports described association with clinical features of PXE such as angioid streaks of the retina and papular skin lesions [2]; PXE is characterized by dystrophic calcification of soft tissues. Patients have normal growth and development [3].

In addition to clinical features, investigations can help establish the diagnosis, especially imaging and histopathological investigations. Vascular calcifications can be revealed as early as during prenatal ultrasound [7]. The preferred imaging modality to assess calcifications extension is whole-body computed tomography [8]. Calcifications can also be detected on routine x-ray films as subtle radiopaque regions. Hence, these findings are easily missed and found by reviewing x-rays, for example the chest x-ray: this pitfall has already been reported [9], and in our case report, has delayed the diagnostic process. Calcifications are also detected, although less frequently, in extravascular sites as periarticular sites [6]. Histopathology, when it is performed, shows diffuse deposition of calcium hydroxyapatite in the internal elastic lamina and diffuse fibrointimal hyperplasia causing patchy luminal narrowing of large- and medium-sized arteries [10].

Our patient’s genetic testing revealed a new familial mutation in the *ENPP1* gene: the unaffected and consanguineous parents were heterozygous carriers for the same mutation as their homozygous son, in accordance with the autosomal recessive mode of inheritance of GACI disease. This mutation, c.784A > G (p.Ser262Gly), is not yet described in the literature and mutation databases. To date, biallelic *ENPP1* mutations are detected in about 70% of all cases of GACI [2] with more than 40 different causative mutations [6]. Most of authors in the literature review don’t have evidence of genotype-phenotype association [11]. Rutsch et al., in a multicenter genetic study and retrospective observational analysis of 55 subjects affected by GACI, have noted one possible genotype-phenotype correlation: homozygosity for the *ENPP1* pathogenic variant p.Pro305Thr was associated with death in infancy in all 5 cases [6]; otherwise, no clear genotype-phenotype correlation was seen in the other subjects of the study.

The *ENPP1* gene encodes an ectonucleotide pyrophosphatase/phopshodiesterase1 ENPP1, which is a membrane-bound enzyme. The ENPP1 enzyme hydrolyzes extracellular ATP in AMP and inorganic pyrophosphate (PPi) [12]. Under physiologic conditions, PPi is a powerful antimineralization factor: PPi inhibits calcification by binding to nascent hydroxyapatite crystals, thereby preventing further growth of the crystals [13]. ENPP1 is the major generator of extracellular PPi, and so plays a key role in regulating vascular calcification process by controlling the levels of circulating PPi. In GACI1, deleterious mutations affecting ENPP1 enzyme restrict PPi generation, leading to propensity for hydroxyapatite deposition [1]. Less frequently, GACI is linked to a mutation in the ATP-binding cassette subfamily C, member6 (ABCC6) gene (GACI2, OMIM 614473) [2] which encodes an ABCC6 [1]. *ABCC6* gene is affected in the classic form of PXE and so, in the same way as there is an overlap in clinical phenotypes, there is a genotypic overlap between GACI and PXE: some patients with GACI harbor mutations in *ABCC6*, and some patients with clinical manifestations consistent with PXE have mutations in *ENPP1* [2]. These observations have suggested the possibility of shared pathomechanistic pathways for these conditions: GACI and PXE represent likely two ends of a clinical spectrum of ectopic calcification and other organ pathologies rather than two distinct disorders [2]. The protein ABCC6 function is unclear: ABCC6 is a putative transmembrane efflux transporter protein expressed primarily in the basolateral plasma membrane of hepatocytes and in the proximal tubules of the kidney; nevertheless, the molecules transported physiologically by ABCC6 from the intracellular milieu to the extracellular space have not been identified yet [1]. ABCC6 has been shown to influence extracellular ATP levels and therefore has a role in the PPi generating pathway. Also, it has been shown that ABCC6 deficiency leads to low extracellular PPi levels [14, 15].

Our patient encountered transient hypophosphatemia: several cases of GACI reported association with hypophosphatemia, and even hypophosphatemic rickets [16]. Hypophosphatemia was found to be associated with a milder phenotype and with a better prognosis [6]. The physiopathology is not well understood. It is hypothesized that hypophosphatemia in GACI might reflect a physiologic compensation mechanism rather than a primary defect. However, a phenotype of autosomal-recessive hypophosphatemic rickets without any arterial calcification was encountered in patients with mutations in the ENPP1 gene, suggesting a different pathway involved in the generation of hypophosphatemic rickets [1].

Due to lack of guidelines, biphosphonates therapy was administered to our patient, as it is the most reported GACI therapy: arterial calcifications were found to decrease (Fig. 1c) but severe refractory hypertension and hypertrophic cardiomyopathy were persistent*.* Data from literature are not consistent*:* in a non-randomized, comparative, retrospective study on few patients (*n* = 43), infants treated with bisphosphonates showed a significantly increased survival [6]. However, both spontaneous resolution of calcifications and fatal outcome despite early therapy have been reported [9, 17]. Hence, it is difficult to determine if bisphosphonates therapy is truly protective or if resolution of calcifications reflects the natural history of the disease. Our patient’s outcome after treatment is consistent with other reports in that resolution of calcifications didn’t correlate with resolution of all cardiovascular findings: it may be a consequence of the persistence of arterial stenosis due to persistent vascular remodelling and damage [6]*.* A better understanding of this rare disease is provided thanks to animal models (such as tiptoe-walking (ttw/ttw) mouse, ages with stiffened joints (asj/asj) mouse and Enpp1 knock-out mouse). New therapeutic strategies are emerging from encouraging animal studies. A recombinant enzyme therapy with subcutaneous administration of an ENPP1-Fc fusion protein was shown to prevent the mortality, vascular calcifications and sequelae in the asj/asj mouse model [18]. Another preclinical work, using *Enpp1*^*asj*^ mice as a model of GACI, demonstrated the dual beneficial effects of bisphosphonates treatment by preventing ectopic mineralization while correcting decreased bone mineralization [19].

Another point to note in this case is the rather favourable outcome: at 2-year-old, the infant presented with severe hypertension but a disappearance of hypertrophic cardiomyopathy. Prognosis in GACI is likely to be poor, most affected individuals die within the first year of life from cardiovascular complications: the mortality rate during the first 6 months of life varies among studies between 85% [10] and 55% for the most recent study [6]. Nevertheless, there are reports in the literature of long-term survival, with several in their 20s [20].

## Conclusion

We report a case of neonatal GACI with three points of interest: the first one is to remind clinicians of this rare and atypical etiology in neonates with severe hypertension and in fetuses with cardiomyopathy and non-immune hydrops fetalis. The second point is the identification of a novel mutation in the *ENPP1* gene associated with a clinical presentation of GACI. The third point is the fairly favourable outcome of our patient after bisphosphonates therapy, with calcifications regression but not hypertension. A better understanding of this rare disease could lead to a better comprehension of more common diseases also associated with calcification impairment (such as chronic kidney disease).

## Abbreviations

ABCC6: ATP-binding cassette subfamily C, member6
asj/asj: ages with stiffened joints mouse
GACI: Generalized Arterial Calcification of Infancy
HCM: hypertrophic cardiomyopathy
PPi: inorganic pyrophosphate
PXE: Pseudoxanthoma Elasticum
ttw/ttw: tiptoe-walking mouse
